# Innovative Use of a Virtual Ward in Managing Severe Neutropenia From Epstein-Barr Virus (EBV)-Associated Mononucleosis: A Case Report

**DOI:** 10.7759/cureus.88403

**Published:** 2025-07-21

**Authors:** Nusrat Ahmed Chowdhury, Shadman Sakib Rahman, Nnaemeka Nnamani, Denis Taiwo

**Affiliations:** 1 Medicine, Medway NHS Foundation Trust, Gillingham, GBR

**Keywords:** epstein-barr virus, granulocyte colony-stimulating factor, immunocompromised patient, infectious mononucleosis, medical and acute response team (smart), remote electronic patient monitoring device, self-isolation, severe neutropenia, surgical, virtual ward

## Abstract

Epstein-Barr virus (EBV)-associated infectious mononucleosis (IM) is a common condition worldwide; however, presenting with severe neutropenia is not something encountered very often. We report such a rare case, where a 19-year-old male patient presented with typical features of mononucleosis and a neutrophil count as low as 0.2 × 10^9^/L. Diagnostic investigations confirmed an EBV infection, while ruling out any hematological or vascular disorder. To reduce the risk of nosocomial infections, it was decided to monitor the patient by remote electronic devices from the comfort of his home under the care of a virtual ward, aiming for full recovery over time. He received his intravenous (IV) antibiotics at home, and alternate-day blood tests showed a gradual and spontaneous rise in his neutrophil count back to baseline levels without any complications. This case highlights the promising role of virtual wards in reducing hospitalization while ensuring patient safety and effective management, particularly when prolonged hospital stay poses added infection risks.

## Introduction

Infectious mononucleosis (IM) is primarily caused by Epstein-Barr virus (EBV), which infects approximately 95% of the healthy population [1]. The infection is spread through the exchange of saliva, and the virus replicates in the oropharynx and remains as a long-lasting dormant infection in the B-cells [2]. A small proportion of affected individuals are also at risk of developing lymphoid or epithelial cell malignancies [2]. Typical features include fever, pharyngitis, lymphadenopathy, and malaise [2]. The condition is usually self-limiting and is classified as a benign lymphoproliferative disorder [3].

It was reported by the International Association for Studying Agranulocytosis and Aplastic Anemia that 4% of the patients can develop agranulocytosis, that is, significantly reduced neutrophil counts up to a year following the acute phase of illness [3]. However, there is limited information about the use of filgrastim (granulocyte colony-stimulating factor (G-CSF)) to induce a rise in neutrophil levels, for it to be considered in the management of EBV-induced agranulocytosis [3].

Agranulocytosis in EBV-associated IM is exceptionally rare, and almost every case is reported [3]. In the last 10 years, only multiple cases of severe leukopenia have been found in the literature [3]. All the cases were reported to be in young individuals with no prior history of any immunocompromised conditions [3].

A study of several such cases found that eight out of nine patients had a rise in neutrophil counts of more than 500/µL within 3-7 days, while two patients developed fatal superimposed bacterial infections within 1-3 days of the onset of neutropenia [4]. Based on these findings, it is evident that severe neutropenia is an infrequent but significant complication of IM and warrants self-isolation, careful monitoring, and early treatment of any secondary infections [4].

## Case presentation

A 19-year-old mixed-race British male patient presented to the Emergency Department with a three-day history of generalized weakness, lethargy, low-grade fever, anorexia, and multiple episodes of non-bilious vomiting. He reports complete inability to tolerate oral intake due to painful oral lesions and sore throat. On further inquiry, he described ulcerative lesions on his tongue and oral mucosa associated with significant odynophagia.

The patient has a recent history of IM, for which he was hospitalized three weeks prior and treated supportively following a positive monospot test. He has no significant past medical history and no known drug allergies and denies any history of illicit drug use. He is sexually active with one female partner and has not used barrier protection. He denies any penile discharge, genital ulcers, or lower urinary tract symptoms.

On examination, his vital signs were within normal limits aside from mild tachycardia (heart rate, 108 bpm). Oral examination revealed multiple whitish ulcerative lesions on the tongue and hard palate. There was marked bilateral tonsillar enlargement (Grade 3) with erythema and exudate, as well as tender bilateral cervical lymphadenopathy. No skin rashes or genital lesions were noted.

Routine blood tests were requested on admission along with blood cultures, sexually transmitted infection (STI) screen (human immunodeficiency virus (HIV), hepatitis B (Hep B)), EBV and cytomegalovirus (CMV) serology, and vasculitis screen (antinuclear antibody (ANA), antineutrophil cytoplasmic antibody (ANCA), extractable nuclear antigen (ENA), double-stranded DNA (dsDNA), complement). The only positive blood result was detecting IgM in the EBV antibody study, confirming an acute EBV infection. The rest of the infection screen was negative, including blood culture (Table 1). The autoimmune panel of investigations was all negative as well (Table 2), which ruled out any autoimmune disease that could occur as a complication of EBV infection. Renal function was normal (Table 3), which ensured any medications, particularly antibiotics, could be safely given at their regular dose according to the patient's body weight. Initial investigation findings revealed a full blood count (FBC) showing a picture of severe neutropenia along with slightly raised monocyte count and raised inflammatory markers (C-reactive protein (CRP)) (Table 4), which prompted screening bloods to be requested to investigate the pathology behind such a low neutrophil count.

**Table 1:** Infection screen to identify the cause of IM IM: infectious mononucleosis; EBV: Epstein-Barr virus; CMV: cytomegalovirus; HBsAg: hepatitis B surface antigen; HIV: human immunodeficiency virus; Hep C Ab: hepatitis C antibody

Test	Results
EBV screen	IgG NOT detected; IgM DETECTED
CMV screen	IgG NOT detected; IgM NOT detected
HBsAg	NOT detected
HIV	NOT detected
Hep C Ab	NOT detected
Blood culture	No growth after five days of incubation

**Table 2:** Vasculitic screen to rule out any other autoimmune disorder that can cause severe neutropenia ANA: antinuclear antibody; ANCA: antineutrophil cytoplasmic antibody; dsDNA: double-stranded DNA; ENA: extractable nuclear antigen

Test	Results
Antinuclear Ab (ANA)	Negative
ANCA	Negative
dsDNA Ab	Negative
ENA Ab Screen	Negative

**Table 3:** Renal function test AKI: acute kidney injury; GFR: glomerular filtration rate

Parameter	Value	Normal range
AKI	Stage 0	
Creatinine (µmol/L)	92	59-104
Estimated GFR	>90	
Potassium (mmol/L)	4.0	3.5-5.3
Sodium (mmol/L)	135	133-146
Urea (mmol/L)	4.7	2.5-7.8

**Table 4:** Full blood count showing trend in neutrophil count over a period of seven days Hb: hemoglobin; WBC: white blood cell; CRP: C-reactive protein

	Day 1	Day 3	Day 5	Day 7	Normal range
Hb (g/dl)	137	138	145	145	130-170
WBC (x10^9^/L)	3.3	7.1	10.7	8.0	4.0-11.0
Neutrophil count (x10^9^/L)	0.2	2.5	6.1	3.9	2.0-7.0
Monocyte count (x10^9^/L)	1.8	1.6	0.8	0.9	<1.0
Lymphocyte count (x10^9^)	1.3	2.9	3.5	3.2	1.0-4.0
Platelet count (x10^9^)	179	205	303	317	150-410
CRP (mg/dl)	220.5	269.7	84.6	16.2	0.0-5.0

An urgent peripheral blood film was requested, and it was found to show marked neutropenia along with few vacuolated monocytes and occasional atypical lymphocytes. Normocytic red cells and raised lymphocyte and monocyte counts were also found, which were most likely to be reactive, as commented by the senior hematologist.

The provisional diagnosis for the patient was EBV-associated IM with severe neutropenia. Other differentials included other viral infections, i.e., CMV or HIV associations, hematological malignancies, and autoimmune disorders, which were tested for and ruled out (Tables 1, 2).

With the following blood results, the patient was shifted from the hospital to the virtual ward after properly explaining to him and his parents about how he will be treated in a home setting under the Surgical, Medical, and Acute Recovery Team (SMART) care. Neutrophil counts were observed on alternate days, and the trend in FBC was observed for seven days. There was a gradual and spontaneous rise of the neutrophil counts from very low levels back to baseline (Table 4).

**Figure 1:**
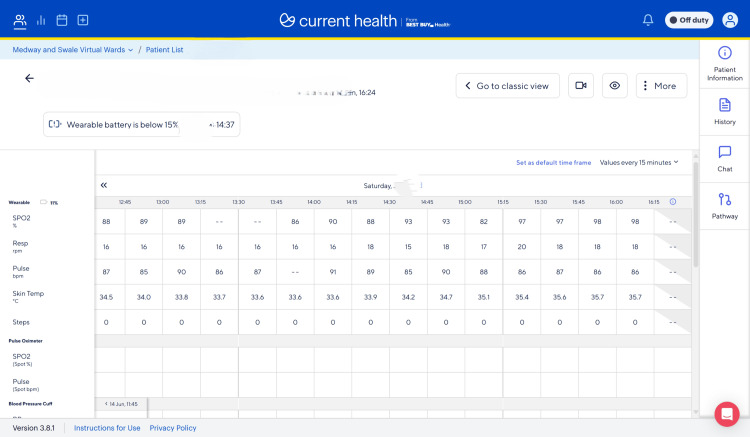
Remote monitoring system dashboard for patients admitted under virtual ward

**Figure 2:**
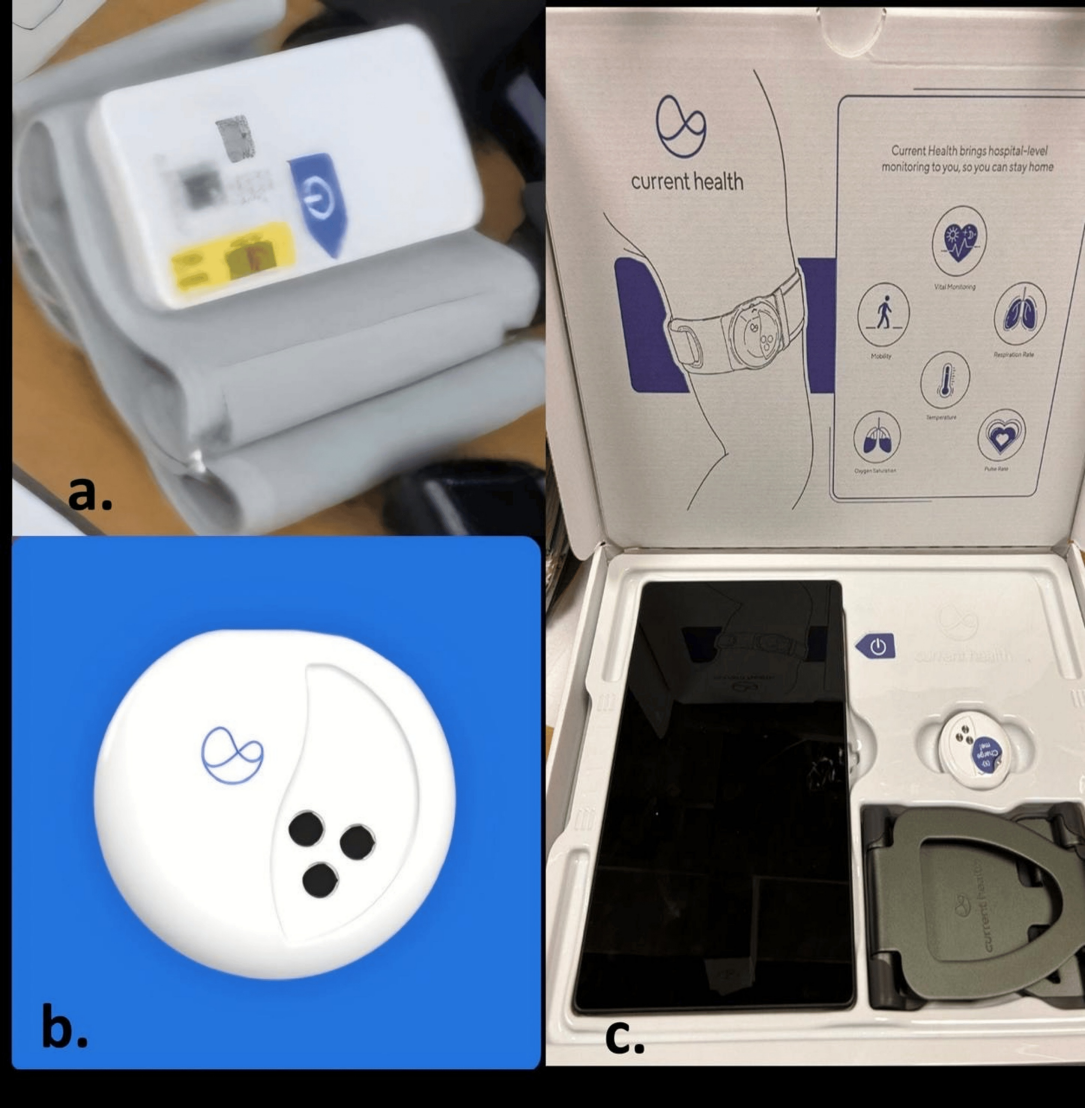
Remote monitoring devices used to monitor patients admitted under virtual ward. (a) Blood pressure cuff, (b) wearable sensor and tracker, (c) vitals monitoring screen

Given the patient's severe neutropenia and the associated increased risk of nosocomial (hospital-acquired) infections, it was deemed clinically appropriate and, in the patient’s best interest, to manage him in a virtual ward setting under the supervision of the SMART team. The patient was thoroughly counselled regarding the rationale for this approach, including the benefits of reducing his exposure to hospital pathogens while still receiving daily clinical oversight and intravenous (IV) antimicrobial therapy. He was commenced on IV ceftriaxone 2 g once daily for seven days and was put on a wearable remote monitoring device (Current Health), which was set to monitor his vital signs every 15-30 minutes initially, until the patient became stable (Figures 1, 2). Antibiotics were used as a precautionary strategy, given the increased risk of secondary bacterial infection in patients with such profound neutropenia. He had alternate-day bloods to monitor neutrophil and inflammatory markers (Table 4). He was encouraged to maintain hydration and oral hygiene with antiseptic mouthwash.

The patient improved clinically, and stable vital signs were maintained through his admission on the virtual ward, as evidenced by the remotely monitoring device system (Figure 1). His neutrophil counts returned to baseline without the need for human G-CSF, and his inflammatory markers were near normal (Table 4). The patient was successfully discharged from the virtual ward and required no in-hospital treatment. The virtual ward offers hospital-level care at the patient’s comfort and convenience at home, using an electronic monitoring device (Figure 2) to monitor the patient remotely while offering the best care.

## Discussion

While mild neutropenia is a common presentation during the first week of illness, seen in around 40% of cases, counts dropping to fewer than 0.2 x 10^9^ polymorphonuclear leukocytes per L are quite rare and not a widely recognized feature [4]. Bone marrow studies during this time showed an increased number of myelocytes and promyelocytes [4]. It is therefore advisable to closely monitor these patients and to provide urgent treatment for any suspected superimposed infection [4].

EBV-specific antibody profiling (Table 1) is considered to be the gold standard for staging of the disease [5]. A study suggested a cycle of infection and reactivation where acute infection produced memory B lymphoblasts (mBLats), which then underwent the natural process of homeostatic apoptosis. Dying mBLats triggered further viral replication [6]. The EBV IgM antibodies in patients presenting with signs and symptoms of IM are likely to be positive for a period of up to 2-3 months since their onset [7]. Usually, IgG appears within the first two weeks and persists lifelong, but in a few cases, it can be delayed by up to 3-4 weeks [8].

Most cases of neutropenia due to viral infections are self-limiting but can be prolonged after an EBV infection [9]. G-CSFs are usually reserved for those who present with severe or recurrent infections, as well as skin infections or mucosal erosion [9].

Virtual ward could play a key role in this situation by ensuring close monitoring of vitals using a remote electronic monitoring device (Figure 2). The remote monitoring device is unique because of its ability to ensure real-time and continuous health monitoring every 30 minutes, one, two, or four hours (Figure 1). It is patient-centred and offers a smooth transition from hospital to the patient’s home, while maintaining an appropriate safety net that enables healthcare professionals to intervene promptly. A virtual ward can be a reliable option for the treatment and monitoring of patients with EBV-associated IM at home. Overall, evidence has shown that with the use of technology and an appropriate safety net in place, virtual wards are safe [10].

Virtual wards are gaining increasing recognition within the NHS and have been effectively utilized to deliver hospital-level care at home for patients who qualify for admission and are comfortable using technology to manage their conditions remotely [11]. The virtual ward is the future of healthcare delivery and a vital part of the NHS system, provided there is a provision of safe monitoring [11].

## Conclusions

While managing EBV-associated IM complicated by severe neutropenia, it is quite crucial to bear in mind the risk associated with keeping them admitted, since they are immunocompromised and susceptible to all the hospital-acquired infections. But it is also quite a challenge to safely discharge them with appropriate antibiotic prophylaxis and close observations, aiming for the spontaneous rise in neutrophil levels along with improvement in clinical conditions. It is important to note the need to avoid prematurely initiating neutropenic sepsis protocols or administering G-CSF without clear indications. In these circumstances, compared to NHS hospital admission, the virtual ward would be an equally effective and safe alternative that is driven by technology.
